# Actinomyces odontolyticus: Rare Etiology for Purulent Pericarditis

**DOI:** 10.1155/2014/734925

**Published:** 2014-12-15

**Authors:** Ryan Mack, Kipp Slicker, Shekhar Ghamande, Salim R. Surani

**Affiliations:** ^1^Department of Internal Medicine, Scott and White Memorial Hospital, 2401 South 31st Street, Temple, TX 76508, USA; ^2^Department of Cardiology, Scott and White Memorial Hospital, 2401 South 31st Street, Temple, TX 76508, USA; ^3^Department of Pulmonary & Critical Care, Scott and White Memorial Hospital, 2401 South 31st Street, Temple, TX 76508, USA

## Abstract

Purulent pericarditis is one of the most common causes of cardiac tamponade and if left untreated has a mortality of 100%. *Staphylococcus aureus* and *Streptococcus pneumonia* have been implicated as the main etiology of purulent pericardial effusion followed by fungi and anaerobic sources. *Actinomyces odontolyticus* pericardial involvement has been reported in the literature only once. To our knowledge, this is the first fatal case of *A. odontolyticus* purulent pericarditis in the absence of periodontal disease.

## 1. Introduction

Purulent pericarditis results in cardiac tamponade in 80% of cases. If left untreated it has a mortality rate of 100% [1]. Pericardial fluid must be obtained through percutaneous or surgical means. Once a sample of pericardial fluid has been obtained, the European Society of Cardiology recommends obtaining gram stain, acid fast smear, fungal smear, pericardial culture, and body fluid cultures [2]. Risks for purulent pericarditis include immunosuppression and chronic alcohol abuse. Typical bacteria causing pericarditis include* Staphylococcus aureus* and* Streptococcus pneumonia*. Fungal and anaerobic sources are rare. Pericardial infection due to* Actinomyces odontolyticus* has been reported in the literature only once [2]. We hereby present a case of 61-year-old male with* Actinomyces odontolyticus* pericardial abscess.

## 2. Case Report

A 61-year-old male presented from his primary care physician's clinic to the emergency department (ED) with a complaint of worsening shortness of breath at rest for the past two days, which was exacerbated by talking. He had associated symptoms of fever, chills, chest pain, and nonproductive cough. His past medical history was significant for stage III, T2N2 metastatic squamous cell lung cancer of the right upper lobe which was diagnosed in 2012. The patient had received three-week cycles of carboplatin and paclitaxel (4 cycles) and then weekly cycles for five weeks. The patient also received concurrent external beam radiation, which was discontinued at patient request after receiving 58 Gy. The patient also was found to have new paratracheal lymph node a year later and one month prior to this presentation on CT scan of chest. The patient underwent endobronchial ultrasound (EBUS) guided needle aspiration of station 7, which was positive for malignancy. The patient had a history of smoking of 90 pack year. The patient was also edentulous and was not wearing dentures.

On examination, the patient was having tachycardia and was hypotensive with pulse of 148 beats per minute and blood pressure of 67/38 mm hg. He was hypoxic with oxygen saturation of 84% on room air, had tachypnea with respiratory rate of 24/min, and was febrile with a temperature of 101.8°F (38.8°C). He was chronically ill in appearance with use of accessory muscles for breathing. Oropharynx was dry with dentures noted. Jugular venous distension was present in the angle of the jaw. Breath sounds were significant for decreased air entry in the right lower base and expiratory wheezes bilaterally. Heart sounds were distant with no murmurs or rubs. There was bilateral pitting edema in his lower extremities.

## 3. Laboratory Findings and Diagnostic Testing

**Figure 1:**
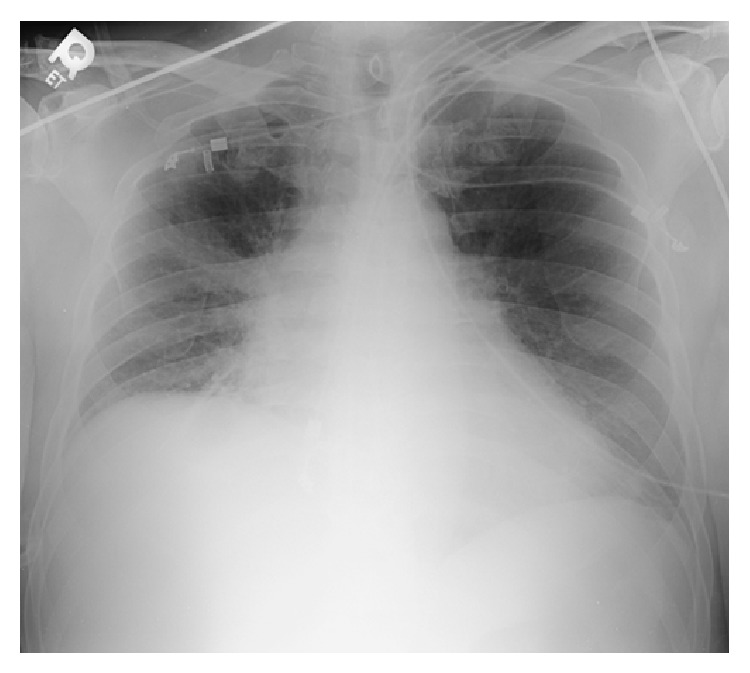
Chest X-ray AP view showing enlarged pericardial silhouette and right hilar mass.

**Figure 2:**
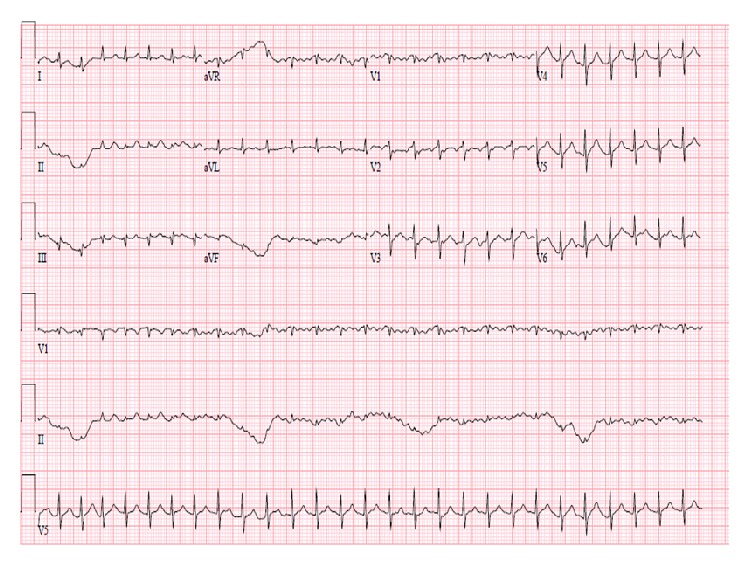
EKG revealing sinus tachycardia with no evidence of pericardial disease.

**Figure 3:**
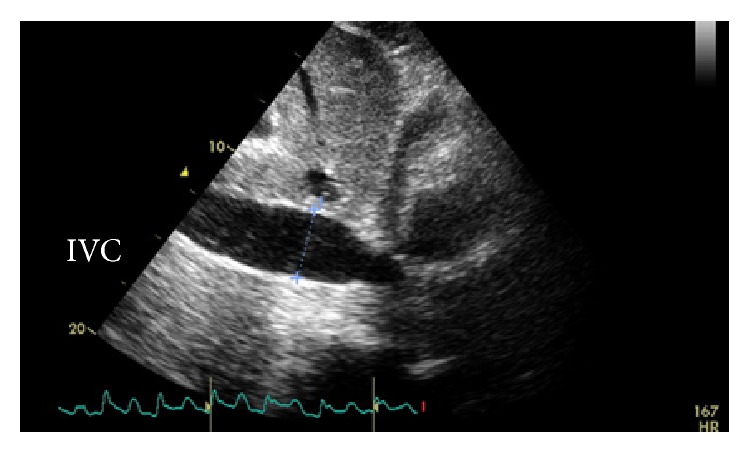
Echocardiographic image showing noncollapsible inferior vena cava with pericardial effusion.

**Figure 4:**
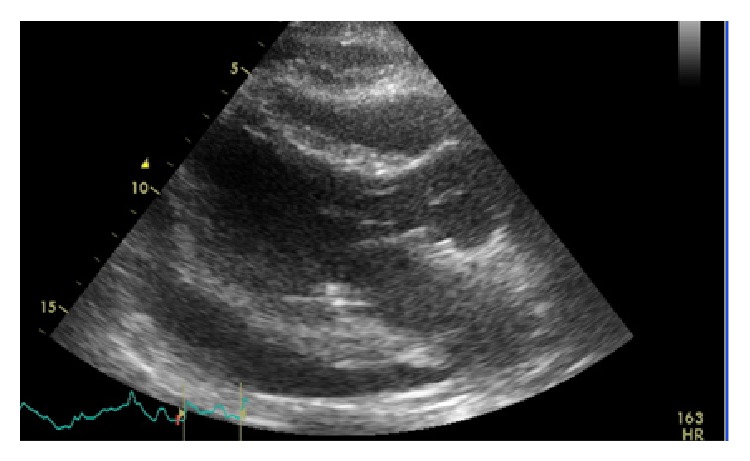
Echocardiographic image showing collapsed right ventricle with effusion.

White blood cell count was elevated to 17,500/mL with a left shift. His comprehensive metabolic profile was unremarkable. Initial troponin and lactate were normal. Initial blood cultures were negative as well. His chest X-ray demonstrated right hilar fullness that was consistent with his known lung cancer and a mildly enlarged cardiac silhouette (Figure 1). EKG on admission was significant for sinus tachycardia with premature atrial complexes as shown in (Figure 2). Following admission to the intensive care unit, the patient had a cardiac arrest secondary to pulseless electrical activity (PEA). He did regain a pulse after 8 minutes of CPR. EKG following this episode revealed low voltage QRS and emergent echocardiography demonstrated moderate circumferential pericardial effusion with right ventricular compression, inferior vena cava dilation, and slight variation with right ventricular size during mechanical respiratory support (Figures 3 and 4). Patient did have severe hypotension and pulsus paradoxus. Given the state of shock, emergent bedside pericardiocentesis was performed and 400 mL of purulent pericardial fluid was removed and pericardial drain was left in place. The gram stain had a large amount of white blood cells and rare gram positive cocci. After three days, the pericardial fluid culture isolated 2 colony forming units of* Actinomyces odontolyticus.* The patient was diagnosed with cardiac tamponade secondary to* Actinomyces odontolyticus* pericarditis in an immunocompetent adult.

Following his cardiac arrest, the patient did have one further episode of ventricular tachycardia requiring cardioversion. He was treated with piperacillin-tazobactam, vancomycin, and ciprofloxacin. Over the following 24 hours, the patient developed disseminated intravascular coagulation and multiorgan failure, with WBC 26,700/mL, AST 27,281, ALT 10,725, lactate of 8.1 mmol/L, and INR of 3.3. A repeat echocardiogram on hospital day 4 showed significant improvement of the pericardial effusion; however, the patient continued to decline clinically. Since there was significant improvement in the pericardial effusion, pericardiectomy or fibrinolysis was not done. The patient remained in refractory shock despite the vasopressor therapies. He was transitioned to comfort care on hospital day 6 with compassionate extubation and died shortly thereafter. No further organisms were cultured from the blood, urine, or sputum or by bronchial lavage.

## 4. Discussion

Drainage of the pericardial fluid is essential in purulent pericarditis. This can be accomplished through a pericardiocentesis, formation of a “pericardial window,” or pericardiectomy. The more invasive procedures required for pericardial window or pericardiectomy are able to achieve improved drainage of the pericardial fluid but do have an associated increase in morbidity and mortality with the procedure itself [2].

Antimicrobial treatment consists of broad spectrum antibiotics initially targeting both gram negative and gram positive species. If the patient is immunosuppressed and high suspicion exists for a fungal source, empiric antifungal treatment should be added as well. Therapy can then be targeted at any bacterial isolates from either the blood or the pericardial cultures. There is also suggestion of use of intrapericardial fibrinolysis for any loculated fluid collections and as an alternative treatment regimen as opposed to complete removal of the pericardium [3, 4].

*Actinomyces odontolyticus* causing invasive disease has been previously reported in 25 patients [1]. It is thought to be related to immunosuppression and is normally seeded from oropharyngeal sources. This patient is the second reported case of pericardial* A. odontolyticus* but the first with exclusive pericardial disease. The source is unclear in this patient given isolation from a typically sterile fluid and from no other sites. There was no suspicion for dental abscess as the patient had previously had all teeth removed and dentures in place. It was unlikely that he was immunocompromised. His induction chemotherapy for his squamous cell lung carcinoma with paclitaxel and carboplatin was completed one year prior to his presentation. This case highlights the pathology that can be induced by* A. odontolyticus* even in the absence of immunosuppression.

## 5. Clinical Pearls

1. A broad differential diagnosis must be considered when approaching purulent pericarditis with consideration of atypical etiologies.
2. Cardiac tamponade is a common complication of purulent pericarditis.
3. Prompt recognition of purulent pericarditis is essential as the mortality in untreated cases can reach 100%.
4. Treatment in purulent pericarditis consists of drainage of pericardial fluid and targeted parenteral antimicrobial therapy. If no prompt recovery is observed, pericardiectomy with extensive rinsing of the purulent effusion remains the recommended treatment [2]. Alternatively, intrapericardial fibrinolysis can be attempted if there are major contraindications or patient is not accepting surgery.
5. We report* A. odontolyticus* causing a fatal purulent pericarditis in an immunocompetent patient.
